# Serious Game–Assisted Teaching for Junior Operating Room Nurses in Unicompartmental Knee Arthroplasty: Quasi-Historical Controlled Trial

**DOI:** 10.2196/93169

**Published:** 2026-07-14

**Authors:** Feng Zhang, Xiao Yan Wang, Li Li Wang, Juan Guo, Li Zhang, Jing Zhang, Ping Wang, Li En Qi

**Affiliations:** 1 Operating Room, Xuzhou Central Hospital, 199 Jiefang South Road, Xuzhou, Jiangsu, 221009, China, 86 18952171220; 2 Department of Nursing, Xuzhou Central Hospital, Xuzhou, Jiangsu, China

**Keywords:** education, simulation, self-efficacy, cognitive, nurses, orthopedics, game

## Abstract

**Background:**

Unicompartmental knee arthroplasty (UKA) requires precise intraoperative coordination from operating room nurses. Conventional teaching may be insufficient for junior nurses learning complex UKA surgical cooperation, particularly regarding self-efficacy and cognitive demands. Serious games have emerged as a promising educational tool for nursing training.

**Objective:**

This study evaluated whether adding a WeChat-based serious game to conventional UKA training was associated with improved theoretical knowledge, self-efficacy, preoperative preparation, intraoperative performance, and perceived cognitive load among junior operating room nurses.

**Methods:**

This quasi-historical controlled study compared 2 sequential, noncontemporaneous cohorts of newly recruited junior operating room nurses from a tertiary hospital in Xuzhou, China. Nurses recruited in 2023 were assigned to the conventional training group (n=21, 50%), whereas nurses recruited in 2024 were assigned to the serious game–assisted training group (n=21, 50%). Both groups received the same UKA curriculum, including theoretical teaching and supervised intraoperative practice, while the intervention group additionally used a mobile serious game. Outcomes included theoretical test scores, General Self-Efficacy Scale scores, National Aeronautics and Space Administration Task Load Index (NASA-TLX) cognitive load scores, and assessments of preoperative preparation and intraoperative performance.

**Results:**

Data from all 42 eligible participants were included. Compared with conventional training, serious game–assisted training was associated with higher theoretical knowledge scores (mean 96.86, SD 3.09 vs mean 92.38, SD 4.32; *P*<.001; Cohen *d*=1.19), preoperative preparedness (mean 4.19, SD 0.33 vs mean 3.49, SD 0.29; *P*<.001; Cohen *d*=2.27), intraoperative performance (mean 4.00, SD 0.30 vs mean 3.41, SD 0.28; *P*<.001; Cohen *d*=2.03), and overall preparedness and performance (mean 4.10, SD 0.23 vs mean 3.45, SD 0.20; *P*<.001; Cohen *d*=3.02). Self-efficacy was higher in the unadjusted analysis (mean 34.05, SD 2.87 vs mean 31.57, SD 3.43; *P*=.02; Cohen *d*=0.78), but this difference was attenuated after adjustment for age and education (adjusted mean difference 2.45, 95% CI −0.26 to 5.15; *P*=.08). The serious game–assisted group also reported higher NASA-TLX total cognitive load (mean 65.44, SD 12.00 vs mean 58.21, SD 6.05; *P*=.02; Cohen *d*=0.76).

**Conclusions:**

Serious game–assisted training was associated with better theoretical knowledge and procedural performance but higher perceived cognitive load among junior operating room nurses learning UKA coordination. The adjusted self-efficacy result, as well as the overall findings, should be interpreted cautiously rather than as definitive causal evidence because the study used a quasi-historical design without a contemporaneous control group.

## Introduction

### Background and Rationale

Instrument nurse cooperation ability is one of the core competencies required for operating room nurses. In the unique environment of the operating room, efficient collaboration with surgeons and other professionals is key to ensuring the smooth progress of surgery [1,2]. Unicompartmental knee arthroplasty (UKA), as a precise surgical procedure for unicompartmental knee disease, offers advantages over total knee arthroplasty, such as less trauma, a shorter recovery period, and better preservation of proprioception and joint function [3]. However, it places stringent demands on the coordination ability of instrument nurses.

Self-efficacy refers to nurses’ belief in their ability to achieve goals and successfully handle difficulties encountered in the process, reflecting their confidence in achieving objectives [4], which may affect their psychological perception, mentality, and behavior [5]. Cognitive load refers to the total mental effort expended in working memory, consisting of intrinsic load (complexity of the task), extraneous load (processes not directly contributing to learning), and germane load (schema construction during learning) [6]. Cognitive work in nursing refers to organization, sequencing, and decision-making in practice, which constitute a nurse’s cognitive load.

Junior nurses commonly face dual challenges in learning UKA surgical coordination. On one hand, they may have limited familiarity with procedural steps, operative anatomy, and intraoperative decision-making, which may reduce confidence and perceived self-efficacy [7,8]. On the other hand, UKA procedures are time-sensitive and technically demanding, requiring rapid instrument switching, sustained attention, and multitask coordination within a limited time frame, which may increase cognitive load and affect procedural performance 8. In this study, these challenges were operationalized through broader educational outcomes, including self-efficacy, perceived cognitive load, and observer-rated preoperative preparation and intraoperative performance, rather than through direct measurement of specific technical subskills.

Currently, clinical nursing training mainly relies on traditional methods such as theoretical lectures and operational demonstrations. However, the separation of theory and practice makes it difficult for nurses to apply what they have learned, resulting in poor learning outcomes. Furthermore, junior nurses’ lack of confidence in performing surgical cooperation tasks and the high cognitive load generated when learning complex UKA procedures further restrict training effectiveness. The one-way teaching model in traditional training tends to increase cognitive fatigue, weaken initiative, and form a vicious cycle of “low self-efficacy–high cognitive load.” The lack of personalized training cannot meet different nurses’ needs, and the underdeveloped feedback and evaluation system hinders understanding of individual progress. Therefore, introducing diverse teaching methods closely integrated with clinical practice is urgently needed to effectively improve junior nurses’ surgical cooperation skills.

In recent years, innovative teaching methods have attracted increasing attention in nursing education. Among them, virtual clinical simulation provides an immersive learning environment, allowing students to practice nursing skills in a safe, controlled setting and simulating complex real-world clinical scenarios [9], thus compensating for the shortcomings of traditional teaching. Serious games—interactive computer applications designed to deliver specific learning objectives—serve as virtual simulation teaching tools. Through realistic simulated environments and risk-free error consequences, learners acquire key skills such as problem analysis, problem-solving, and teamwork through role-playing, thereby enhancing motivation, engagement, and experiential learning [10]. Mitchell et al [11] explored the impact of serious games on nursing students’ knowledge and behavior regarding influenza vaccination, confirming the potential value of serious games in nursing practice and behavior change. Aloweni et al [12] further demonstrated that serious games not only have substantial educational potential but also effectively enhance nurses’ professional knowledge and skills.

### Study Aim

Therefore, the aim of this study was to evaluate whether adding a WeChat-based serious game to conventional UKA training for junior operating room nurses was associated with improved theoretical knowledge, self-efficacy, preoperative preparation, and intraoperative performance, and with differences in perceived cognitive load compared with conventional training alone. Given the quasi-historical design, the findings were interpreted cautiously because temporal changes in the clinical setting could not be fully controlled.

## Methods

### Study Design

This study was a completed single-center quasi-historical controlled educational study that compared 2 sequential cohorts of junior operating room nurses involved in UKA coordination. In this context, “quasi-historical” indicates that the comparison was made between 2 sequential cohorts recruited in different calendar years rather than between concurrently allocated groups. The analysis was based on deidentified training and assessment data from 2 consecutive nurse entry cohorts at Xuzhou Central Hospital: nurses recruited in 2023 received conventional training, whereas nurses recruited in 2024 received serious game–assisted training. To strengthen internal validity in this quasi-historical comparison, we applied identical eligibility criteria, used the same clinical teaching team, and maintained the same training schedule, curriculum content, and assessment standards across both cohorts. All trainees received the same UKA curriculum, supervised intraoperative mentoring (12 assisted UKA cases), and standardized evaluations using the same scoring rubrics. However, because the comparison relied on a historical control design rather than contemporaneous allocation, temporal confounding cannot be fully excluded. Year-to-year differences in staffing, workflow, case exposure, teaching context, or other unmeasured environmental factors may have contributed to the observed between-group differences. Therefore, baseline characteristics were compared between groups, and sensitivity analyses adjusting for age and education were performed.

### Ethical Considerations

Ethics approval for the research use and analysis of deidentified training and assessment data was obtained from the Xuzhou Central Hospital Biomedical Research Ethics Review Committee on February 26, 2025 (XZXY-LJ-20250226‐032). The study was conducted in accordance with the Declaration of Helsinki and institutional requirements for medical research involving human participants. Written informed consent was obtained from all participants before the research use and analysis of their training and assessment data. All data were deidentified before analysis, and no personally identifiable information was reported in this manuscript or the supplementary materials. Participants received no compensation for inclusion in this analysis.

### Trial Registration

This study was not prospectively registered because it was a quasi-historical controlled educational study rather than a randomized controlled trial. The study protocol, eligibility criteria, training procedures, and outcome assessments were defined before data collection and were implemented consistently across the 2 cohorts.

### Participants

A total of 42 newly recruited junior operating room nurses from Xuzhou Central Hospital were included in the study. The 2023 entry cohort comprised 21 (50%) nurses and served as the traditional training group, whereas the 2024 entry cohort comprised 21 (50%) nurses and served as the serious game–assisted training group. Baseline characteristics were descriptively summarized for the 2 groups.

The inclusion criteria included being newly recruited nurses; having no prior operating room work experience before employment; participating in theoretical training, skills training, and operative cooperation practice related to UKA; and being informed and willing to participate in the study.

The exclusion criteria included being unable to participate in orthopedic surgery cooperation due to pregnancy or other special reasons; being on sick leave, maternity leave, or not continuously employed during the study period; or missing more than 3 training sessions for any reason during the study period.

### Instruments

#### Baseline Sociodemographic and Occupational Characteristics

Baseline sociodemographic and occupational characteristics were extracted from deidentified training and personnel records and included sex, age, educational background, marital status, and professional title. These variables were summarized descriptively.

#### Outcome Measures

##### Theoretical Performance, Cognitive Load, and Self-Efficacy

Because the study focused on overall educational and procedural performance, outcomes were assessed using self-efficacy, cognitive load, and observer-rated preparation and intraoperative performance rather than direct task-specific measures, such as instrument transfer accuracy or anatomic identification.

A self-designed theoretical knowledge questionnaire was used, with a total score of 100 and a passing score of 80. The full text of the self-designed theoretical knowledge questionnaire is provided in Multimedia Appendix 1.

Cognitive load was assessed using the National Aeronautics and Space Administration Task Load Index (NASA-TLX) developed by Hart and Staveland [13]. The instrument includes 6 dimensions, each represented by 1 rating item: mental demand, physical demand, temporal demand, performance, effort, and frustration. Each dimension was rated on a scale from 0 to 20, with higher scores indicating greater perceived task load for that dimension. In accordance with the scoring approach used in the Chinese version reported by Liang et al [14], the overall NASA-TLX score was calculated as a weighted composite score (TLX=∑[ρi×ri]), with a total range of 0 to 100. Higher total scores indicate greater perceived cognitive load. The Chinese version has shown acceptable reliability, with a split-half reliability of 0.808 and a Cronbach α of 0.758 [14].

Self-efficacy was assessed using the General Self-Efficacy Scale (GSES), which contains 10 items scored from 1 to 4, yielding a total score of 10 to 40. Higher scores indicate higher self-efficacy [15].

##### Preoperative Preparation and Intraoperative Performance

These assessment items were directly adopted from the questionnaire reported by Zhao and Cong [1] and were used without modification. Senior nurses completed the ratings after the operation using a 5-point Likert scale (1=strongly disagree; 5=strongly agree).

##### Feedback on Training Methods

The posttraining feedback questionnaire was directly adopted from the instrument reported by Zhao and Cong [1] and was used without modification. The questionnaire consists of 10 items rated on a 5-point Likert scale and was completed by junior nurses after training in both groups. In the primary analysis, these items were used as posttraining feedback measures for between-group comparison. Details of the serious game design, interface, gameplay workflow, and representative screenshots are provided in Multimedia Appendix 2.

### Training Programs

The control group received traditional teaching consisting of two stages: (1) 2 weeks of theoretical study (PowerPoint lectures and on-site simulation) and (2) 4 weeks of intraoperative one-on-one mentoring, with each nurse completing 12 UKA cooperation sessions under supervision.

In the game teaching group, a mobile game (“Joint Replacement Master”) was introduced in addition to the traditional teaching methods. After the first stage, nurses were instructed in the operation of the game. During the second stage, nurses played the game 3 times before surgery each day to reinforce their understanding of UKA steps.

The game, inspired by WeChat’s “3D Puzzle,” features 29 levels corresponding to UKA steps. Instruments are integrated into a “tool library.” Realistic videos simulate the surgical environment. Each level provides step prompts; correct instrument selection progresses the game, whereas errors prompt immediate feedback and require a retry. The game includes a story format, badge rewards, background music, and a ranking system to enhance engagement and competition.

Details of the serious game design, interface, gameplay workflow, and representative screenshots are provided in Multimedia Appendix 2.

### Statistical Analysis

Descriptive statistics (frequency, mean, SD, and range) were calculated. Differences between groups were analyzed using 2-tailed independent-sample *t* tests for continuous variables, chi-square tests for categorical variables, and Mann-Whitney *U* tests for ordinal variables. Statistical significance was set at *P*<.05. All analyses were performed using SPSS (version 27.0; IBM Corp).

In addition to *P* values, measures of practical importance were reported for key between-group comparisons, including mean differences (MDs) with 95% CIs, Cohen *d*, and eta-squared, to facilitate interpretation of the magnitude of the observed effects. Test statistics and degrees of freedom were reported where appropriate.

As a robustness check, we fitted analyses. of covariance and linear regression models with the posttraining outcome as the dependent variable and group (game vs traditional) as the primary predictor, adjusting for age (continuous) and education level (categorical). Given the small sample size, HC3 robust SEs were used to estimate adjusted MDs (β for group) with 95% CIs.

## Results

### Participant Cohorts and Analytical Sample

This manuscript reports a completed analysis of 2 sequential cohorts of newly recruited junior operating room nurses at Xuzhou Central Hospital. The traditional training cohort was recruited in 2023 (n=21), and the serious game–assisted training cohort was recruited in 2024 (n=21). Complete training and outcome assessment data were available for all 42 eligible participants, and the final statistical analyses were completed before manuscript submission. Because the study compared noncontemporaneous cohorts recruited in 2023 and 2024, all between-group findings should be interpreted with awareness of potential temporal confounding.

### Baseline Sociodemographic and Occupational Characteristics

Table 1 presents the baseline sociodemographic and occupational characteristics of the 2 cohorts. Overall, the distributions of sex, age, educational background, marital status, and professional title were broadly similar between the conventional training and serious game–assisted training groups.

**Table 1.** Sociodemographic and occupational characteristics of the 2 groups of nurses (N=42)^a^.

Characteristics	Conventional training group (n=21)	Serious game–assisted training group (n=21)
Sex, n (%)		
Male	6 (29)	9 (43)
Female	15 (71)	12 (57)
Age (y), mean (SD)	22.52 (1.75)	22.38 (2.33)
Educational background, n (%)		
Junior college	9 (43)	6 (29)
Undergraduate student	12 (57)	11 (52)
Graduate student	0 (0)	4 (19)
Marital status, n (%)		
Unmarried	21 (100)	21 (100)
Married	0 (0)	0 (0)
Professional title, n (%)		
Nurse	20 (95)	17 (81)
Nurse practitioner	1 (5)	4 (19)

a Percentages are shown without decimal places because the group sample sizes were less than 100. Baseline characteristics are presented descriptively; no baseline significance testing was performed.

### Theoretical Performance, Cognitive Load, and Self-Efficacy

Table 2 summarizes between-group comparisons with effect sizes. After training, the serious game–assisted training group had higher theoretical performance scores than the conventional training group (mean 96.86, SD 3.09 vs mean 92.38, SD 4.08; *t*_40_=4.009; *P*<.001). The between-group difference in theoretical performance also showed a large effect size (Cohen *d*=1.24; η²=0.287).

**Table 2.** Comparison of theoretical performance, cognitive load, and self-efficacy between the 2 groups after training.

Outcome	Conventional training group (n=21), mean (SD)	Serious game–assisted training group (n=21), mean (SD)	Mean difference^a^ (95% CI)	Cohen *d* (95% CI)	η^2^	*t* test (*df*)	*P* value
Theoretical performance	92.38 (4.32)	96.86 (3.09)	4.48 (2.14 to 6.82)	1.19 (0.53 to 1.85)	0.272	3.864(40)	<.001
Self-efficacy	31.57 (3.43)	34.05 (2.87)	2.48 (0.50 to 4.45)	0.78 (0.15 to 1.41)	0.139	2.537 (40)	.02
NASA-TLX^b^ total score	58.21 (6.05)	65.44 (12.00)	7.23 (1.30 to 13.16)	0.76 (0.13 to 1.38)	0.132	2.465 (40)	.02
Mental demand	10.13 (4.75)	15.45 (9.74)	5.32 (0.54 to 10.10)	0.69 (0.07 to 1.31)	0.112	2.252 (40)	.03
Physical demand	8.87 (4.78)	8.17 (6.89)	−0.70 (−4.40 to 3.00)	−0.12 (−0.72 to 0.49)	0.004	−0.382 (40)	.71
Temporal demand	8.72 (6.89)	13.71 (8.45)	4.99 (0.18 to 9.80)	0.65 (0.02 to 1.26)	0.099	2.096 (40)	.04
Frustration	10.93 (6.74)	11.58 (7.44)	0.65 (−3.78 to 5.08)	0.09 (−0.51 to 0.70)	0.002	0.296 (40)	.77
Effort	8.83 (4.22)	7.03 (6.68)	−1.81 (−5.29 to 1.68)	−0.32 (−0.93 to 0.29)	0.027	−1.047 (40)	.30
Performance	10.72 (6.96)	9.50 (7.08)	−1.23 (−5.61 to 3.15)	−0.18 (−0.78 to 0.43)	0.008	−0.567 (40)	.57

a Mean differences were calculated as serious game–assisted training minus conventional training.

b NASA-TLX: National Aeronautics and Space Administration Task Load Index.

For cognitive load (NASA-TLX), the game group reported higher total scores (mean 65.44, SD 12.00 vs mean 58.21, SD 6.05; *t*_40_=2.465; *P*=.02), indicating higher perceived workload. Specifically, the game group reported higher mental demand (mean 15.45, SD 9.74 vs mean 10.13, SD 4.75; *t*_40_=2.252; *P*=.03) and temporal demand (mean 13.71, SD 8.45 vs mean 8.72, SD 6.89; *t*_40_=2.096; *P*=.04). No between-group differences were observed in physical demand (*P*=.71), frustration (*P*=.77), effort (*P*=.30), or performance (*P*=.57).

After adjusting for age and education (analysis of covariance and linear regression with HC3 robust SEs), the between-group differences remained consistent for theoretical performance (adjusted MD 3.94, 95% CI 1.04-6.83; *P*=.008) and NASA-TLX total scores (adjusted MD 6.97, 95% CI 0.17-13.78; *P*=.045).

### Preparation and Intraoperative Performance

As shown in Table 3, the serious game–assisted group generally scored higher than the conventional training group on the preoperative preparedness and intraoperative performance items. The average score for preoperative preparation was significantly higher in the game group (mean 4.19, SD 0.33 vs mean 3.49, SD 0.29; *P*<.001), as was intraoperative performance (mean 4.00, SD 0.30 vs mean 3.41, SD 0.28; *P*<.001), and the overall average score (mean 4.10, SD 0.23 vs mean 3.45, SD 0.20; *P*<.001).

**Table 3.** Preparedness and performance feedback statement mean scores.

Feedback questionnaire statement	Serious game–assisted training group (n=21), mean (SD)	Conventional training group (n=21), mean (SD)	Mean difference^a^ (95% CI)	Cohen *d* (95% CI)	η^2^	*P* value
Preoperative preparedness						
I felt that the nurse checked the surgical instruments before starting the procedure	4.14 (0.48)	3.57 (0.51)	0.57 (0.26-0.88)	1.16 (0.50-1.81)	0.261	<.001
The nurse knew and understood the relevant anatomy	4.14 (0.57)	3.43 (0.68)	0.71 (0.32-1.11)	1.14 (0.48-1.79)	0.254	<.001
The nurse knew the operation procedure steps	4.24 (0.54)	3.52 (0.60)	0.71(0.36-1.07)	1.25 (0.58-1.91)	0.291	<.001
The nurse was well aware of the operation procedure specific details that could have affected or changed the procedural steps or decisions	4.24 (0.54)	3.29 (0.56)	0.95 (0.61-1.30)	1.73 (1.01-2.44)	0.441	<.001
Overall, the nurse was well prepared for the operation procedure	4.19 (0.51)	3.62 (0.74)	0.57 (0.17-0.97)	0.90 (0.26-1.53)	0.175	.006
Preoperative preparedness score	4.19 (0.33)	3.49 (0.29)	0.70 (0.51-0.90)	2.27 (1.48-3.05)	0.576	<.001
Intraoperative performance						
The nurse’s performance was not affected by stress or anxiety	4.10 (0.44)	3.33 (0.58)	0.76 (0.44-1.08)	1.49 (0.79-2.17)	0.368	<.001
The nurse was insightful and understood the surgeon’s directions	4.00 (0.63)	3.43 (0.60)	0.57 (0.19-0.96)	0.93 (0.29-1.56)	0.185	.005
The nurse had a better grasp of key procedure steps	3.95 (0.59)	3.43 (0.75)	0.52 (0.10-0.94)	0.78 (0.15-1.40)	0.137	.02
The nurse delivered the right tool according to the procedure	4.05 (0.67)	3.43 (0.51)	0.62 (0.25-0.99)	1.04 (0.39-1.68)	0.222	.002
The nurse was a good assistant to the operation	3.90 (0.54)	3.43 (0.75)	0.48 (0.07-0.88)	0.73 (0.10-1.35)	0.123	.02
Intraoperative performance score	4.00 (0.30)	3.41 (0.28)	0.59 (0.42-0.78)	2.03 (1.27-2.77)	0.519	<.001
Overall preparedness and performance score	4.10 (0.23)	3.45 (0.20)	0.65 (0.51-0.78)	3.02 (2.11-3.90)	0.705	<.001

a Mean differences were calculated as serious game–assisted training minus conventional training.

Adjusted analyses also supported higher preoperative preparedness (adjusted MD 0.65, 95% CI 0.42-0.89; *P*<.001), intraoperative performance (adjusted MD 0.61, 95% CI 0.38-0.83; *P*<.001), and overall performance (adjusted MD 0.63, 95% CI 0.47-0.79; *P*<.001) in the game group; the adjusted between-group difference in self-efficacy was attenuated (adjusted MD 2.45, 95% CI –0.26 to 5.15; *P*=.08).

### Feedback on Training Methods

According to Table 4, the game group scored higher on most feedback questionnaire items. Overall satisfaction with the training method was significantly higher in the game group (mean 4.06, SD 0.17 vs mean 3.44, SD 0.20; *P*<.001). The game group also rated the method as better designed (*P*<.001), more conducive to achieving learning objectives (*P*<.001), more helpful for skill improvement (*P*=.03), more interesting (*P*<.001), and more likely to enhance judgment (*P*=.02) and satisfaction (*P*<.001). However, there was no significant difference between groups in confidence improvement (*P*=.16).

**Table 4.** Posttraining feedback questionnaire scores for the 2 training methods.

Item	Serious game–assisted training group (n=21), mean (SD)	Conventional training group (n=21), mean (SD)	Mean difference^a^, 95% CI	Cohen *d* (95% CI)	η^2^	*P* value
Training method was well designed	4.19 (0.51)	3.29 (0.64)	0.90 (0.54 to 1.27)	1.56 (0.85 to 2.24)	0.389	<.001
Training method achieved our learning goals	4.10 (0.54)	3.14 (0.65)	0.95 (0.58 to 1.33)	1.59 (0.88 to 2.28)	0.398	<.001
Teaching method made me confident	3.81 (0.51)	3.52 (0.75)	0.29 (–0.11 to 0.69)	0.45 (–0.17 to 1.06)	0.049	.16
Training method was useful to improve operation nursing skills	3.90 (0.62)	3.48 (0.60)	0.43 (0.05 to 0.81)	0.70 (0.07 to 1.32)	0.114	.03
Training method stimulated my interest	4.14 (0.65)	3.38 (0.59)	0.76 (0.37 to 1.15)	1.22 (0.56 to 1.88)	0.282	<.001
Training method was effective	4.00 (0.55)	3.52 (0.51)	0.48 (0.15 to 0.81)	0.90 (0.26 to 1.53)	0.175	.006
I accomplished complete understanding of the operation procedure	3.90 (0.62)	3.24 (0.54)	0.67 (0.30 to 1.03)	1.14 (0.48 to 1.79)	0.255	<.001
I developed individual judgment	4.10 (0.62)	3.62 (0.67)	0.48 (0.07 to 0.88)	0.74 (0.11 to 1.36)	0.124	.02
I would like to repeat the experience	4.14 (0.48)	3.52 (0.51)	0.62 (0.31 to 0.93)	1.25 (0.58 to 1.91)	0.291	<.001
The overall satisfaction of nurses with the method	4.29 (0.46)	3.67 (0.48)	0.62 (0.32 to 0.91)	1.31 (0.63 to 1.97)	0.310	<.001
Total score	4.06 (0.17)	3.44 (0.20)	0.62 (0.50 to 0.73)	3.38 (2.42 to 4.33)	0.750	<.001

a Mean differences were calculated as serious game–assisted training minus conventional training.

## Discussion

### Principal Findings

This study found that, compared with conventional training alone, serious game–assisted training was associated with higher theoretical knowledge scores, better preoperative preparation, and better intraoperative performance among junior operating room nurses involved in UKA coordination. The serious game–assisted group also showed higher self-efficacy in unadjusted analyses, although this between-group difference was attenuated after adjustment for age and education. In addition, participants in the serious game–assisted group reported higher overall cognitive load, particularly in the domains of mental demand and temporal demand. Taken together, these findings suggest that serious game–assisted training may be a useful adjunct to conventional teaching for improving learning and procedural performance in this setting, while also increasing perceived cognitive demands.

Importantly, the interpretation was strengthened by reporting practical significance indices in addition to *P* values. The effect sizes indicated large differences for theoretical performance (Cohen *d*=1.24), preoperative preparedness (Cohen *d*=2.27), intraoperative performance (Cohen *d*=2.03), and overall preparedness and performance (Cohen *d*=3.02), and a moderate-to-large difference for NASA-TLX total cognitive load (Cohen *d*=0.76). These findings suggest that the observed differences were not only statistically detectable but also meaningful in educational practice. In contrast, the interpretation of self-efficacy remained cautious because the adjusted analysis attenuated this difference.

### Comparison With Prior Work

Practical training is a cornerstone of nursing education, yet the transition from student to professional operating room nurse often requires substantial time, repeated exposure, and progressive familiarity with complex procedural tasks [16]. In recent years, game-based and simulation-based educational approaches have received increasing attention as potential tools to enhance procedural learning and learner engagement. A meta-analysis suggested that serious games may improve learner interaction, motivation, and educational outcomes in nursing education [17].

A 2024 systematic review and meta-analysis of randomized controlled trials reported that digital serious games were associated with improvements in nursing students’ knowledge, performance, and confidence, although heterogeneity and limited long-term follow-up required cautious interpretation [18]. Similarly, a 2024 randomized wait-list controlled trial found that a serious smartphone game improved theoretical knowledge in adult basic life support among nursing students, although improvement in practical skills was less consistent [19]. A 2025 systematic review also summarized that serious games in nursing education have been used to enhance knowledge, clinical decision-making, practical skills, teamwork, and learner engagement [20]. Similarly, previous studies have reported that game-based mobile apps were associated with improved knowledge and skills in areas such as extracorporeal membrane oxygenation pipeline preflushing and venous blood specimen collection [21,22]. Our findings are broadly consistent with this emerging literature and extend it to the context of UKA nursing coordination, where rapid instrument switching, familiarity with procedural steps, and efficient teamwork are especially important.

The better preoperative preparation and intraoperative performance observed in the serious game–assisted group may reflect the value of repeated, structured, stepwise practice in reinforcing procedural memory and improving familiarity with key steps of UKA coordination. In the current intervention, the level-based design of the game corresponded to actual procedural steps, and immediate feedback was provided after incorrect choices. Such features may help learners consolidate task sequences, identify weak points, and rehearse decision-making in a low-risk environment before entering the operating room. In this sense, the intervention may have supported transfer from cognitive rehearsal to procedural performance, which is particularly relevant for junior nurses who are still developing confidence and workflow fluency.

### Interpretation of Cognitive Load Findings

At the same time, the higher cognitive load reported in the serious game–assisted group should be interpreted cautiously. The increase was mainly observed in mental demand and temporal demand rather than in frustration, physical demand, or effort. One possible explanation is that the serious game required learners to process procedural cues actively, make rapid judgments, and respond to task demands in a more concentrated way than conventional learning alone. This pattern may indicate greater task engagement and attentional investment during training. However, this study design did not allow us to determine whether the increased cognitive load represented a beneficial challenge, a temporary burden, or some combination of both.

From the perspective of cognitive load theory, structured repetition, immediate feedback, and staged progression may support schema construction in complex learning tasks [23,24]. However, without longitudinal measurement, it would be inappropriate to conclude that the increased cognitive load observed here was necessarily adaptive or transient. Our findings therefore suggest that serious game–assisted teaching may involve a trade-off: better knowledge and performance may occur alongside greater perceived cognitive demands during training. Future studies should examine how this pattern evolves over time, whether cognitive load decreases with repeated exposure, and whether optimal game difficulty can be calibrated to balance challenge and usability.

### Self-Efficacy and Learner Experience

The serious game–assisted group showed higher self-efficacy in unadjusted analyses, but this difference was attenuated after adjustment for age and education (adjusted MD 2.45, 95% CI –0.26 to 5.15; *P*=.08). This finding suggests that the intervention may have had a positive association with perceived confidence, but the strength and independence of this effect remain uncertain. It is possible that repeated game-based rehearsal and greater familiarity with the operative sequence contributed to this pattern. However, given the adjusted analysis, this interpretation should remain cautious.

Feedback data nevertheless indicated that participants in the serious game–assisted group generally rated the training method more favorably than those in the conventional group. They reported higher satisfaction and stronger perceptions that the method was interesting, helpful, and well designed. This is consistent with the broader literature suggesting that interactive and gamified educational methods may enhance learner acceptance and engagement [17,21,22]. In this study, these favorable perceptions are important because they may support adherence to training and willingness to repeat practice. Future studies may incorporate objective back-end use data, such as the number of sessions, completion time, and error patterns, to better understand how serious game use relates to learning outcomes.

### Practical Implications

These findings have practical implications for perioperative nurse training. UKA coordination requires not only declarative knowledge but also procedural sequencing, timely instrument selection, and coordinated interaction with the surgeon. These are difficult competencies to develop through didactic teaching alone. A mobile serious game may offer a feasible supplementary format for repeated preclinical rehearsal, especially when opportunities for direct exposure are limited or when junior nurses are at an early stage of skill acquisition.

The intervention used in this study was delivered through a WeChat-based platform, which may also increase accessibility and ease of implementation in routine clinical education settings. From an operational perspective, such tools may be particularly useful for fragmented learning, reinforcement of procedural steps, and standardization of teaching content across trainees. However, their use should complement rather than replace direct supervision, clinical mentoring, and real-world operative experience.

### Limitations

Several limitations should be acknowledged. First, this study used a quasi-historical controlled design based on sequential cohorts recruited in 2023 and 2024 rather than contemporaneous allocation. Although we used the same eligibility criteria, teaching team, curriculum framework, training duration, and assessment standards across the 2 cohorts, residual temporal confounding cannot be excluded. Year-to-year changes in the clinical setting, such as differences in staffing patterns, case exposure, workflow organization, informal teaching environment, or broader departmental changes, may have influenced learning opportunities and performance independently of the intervention itself. Therefore, the observed between-group differences should be interpreted cautiously and should not be taken as definitive evidence that the intervention alone caused the observed effects.

Second, the study was conducted at a single center with a relatively small sample size, which limits statistical power and generalizability. Third, outcomes were assessed immediately after training, and long-term retention, skill transfer, and sustainability of benefit were not evaluated. Fourth, several outcomes relied on observer-based assessments completed by senior nurses. Although direct observation of performance is a strength of the study, we did not collect or report sufficiently detailed information on the observers, including the number of evaluators involved, whether the same evaluators assessed both groups, or whether formal observer training and calibration procedures were implemented. Interrater reliability was also not formally assessed. As a result, detection bias and variability in scoring cannot be fully excluded. In addition, although the feedback and performance assessment questionnaires were adopted from a previously published source and used without modification, we did not perform additional psychometric testing, such as internal consistency or interrater reliability analysis, in the current sample.

Fifth, although the introduction highlights clinically relevant challenges, such as instrument transfer accuracy, understanding of operative anatomy, and response to intraoperative events, these specific competencies were not directly measured using objective task-based assessments. Instead, the study evaluated broader outcomes, including self-efficacy, perceived cognitive load, and observer-rated preparation and performance. Therefore, the findings should be interpreted as reflecting overall educational and procedural performance rather than direct evidence of improvement in specific technical subskills.

Finally, although the serious game platform is technically capable of exporting objective use data, such back-end logs were not available in this dataset. As a result, engagement was evaluated using questionnaire-derived proxy measures rather than direct behavioral metrics.

### Conclusions

Serious game–assisted teaching was associated with better theoretical performance, preoperative preparation, and intraoperative performance among junior operating room nurses learning UKA coordination, while also being associated with higher perceived cognitive load. These findings support the potential value of serious games as a supplementary educational strategy in perioperative nursing training.

However, because of the quasi-historical design and absence of a contemporaneous control group, the results should be interpreted cautiously and not as definitive causal evidence. Larger, prospective, multicenter studies with objective use metrics and longitudinal follow-up are needed to better establish the effectiveness, mechanisms, and sustainability of serious game–based training in surgical nursing education.

## Supplementary material

10.2196/93169 Multimedia Appendix 1 Unicompartmental knee arthroplasty (UKA) surgical coordination theory questionnaire.

10.2196/93169 Multimedia Appendix 2 Serious game design, workflow, and representative screenshots.

## Abbreviations

GSES: General Self-Efficacy Scale
MD: mean difference
NASA-TLX: National Aeronautics and Space Administration Task Load Index
UKA: unicompartmental knee arthroplasty
