# Supplementary material for: Serious Game–Assisted Teaching for Junior Operating Room Nurses in Unicompartmental Knee Arthroplasty: Quasi-Historical Controlled Trial
Source: JMIR Serious Games. 2026 Jul 14;14:e93169. doi: 10.2196/93169 (PMC13367756; doi:10.2196/93169)
Supplement: Multimedia Appendix 1 [file games-v14-e93169-s001.docx]

**Multimedia Appendix 2.**

Unicompartmental Knee Arthroplasty (UKA) Surgical Coordination Theory Questionnaire

Total Score: 100 points (20 Multiple Choice Questions, 3 points each = 60 points; 20 Fill-in-the-Blank Questions, 2 points each = 40 points)

Name: _________________ Employee ID: _________________

Department: _________________ Score: _________________

**Instructions**

1. This questionnaire assesses theoretical knowledge related to Unicompartmental Knee Arthroplasty (UKA) surgical coordination.

2. Each multiple-choice question has only one best answer. Please write the correct option in the parentheses before the question number.

3. For fill-in-the-blank questions, please provide the surgical procedure steps, instrument names, and key technical points as required.

**Part I: Multiple Choice Questions (20 questions, 3 points each, total 60 points)**

1. ( ) During UKA, after measuring and determining the femoral component size, the scrub nurse should prioritize preparing:

A. Multiple adjacent size trial components

B. The confirmed corresponding size femoral component

C. Any size trial spacer

D. The final tibial plateau insert

2. ( ) The first cut in the tibial resection stage is typically performed using:

A. Sagittal saw

B. Small oscillating saw

C. Reciprocating saw

D. Curved osteotome

3. ( ) The second cut in the tibial resection stage is typically performed using:

A. Small oscillating saw

B. Reciprocating saw

C. Rotary burr

D. Manual starter

4. ( ) After removing the tibial cutting guide, the next critical step is:

A. Install the tibial trial component

B. Perform femoral drilling

C. Perform secondary reaming/grinding

D. Drill cement holes

5. ( ) After femoral drilling is completed, the step that prepares for entering the femoral canal is:

A. Posterior condyle resection

B. Manual canal opening

C. Install trial condyle

D. Measure extension gap

6. ( ) After manual canal opening, the next operation is:

A. Insert the intramedullary rod

B. Secondary reaming/grinding

C. Remove osteophytes

D. Install the final prosthesis

7. ( ) One of the main purposes of the "Femoral Drilling and Alignment" step is:

A. Determine the extension gap

B. Provide a reference for subsequent femoral resection

C. Remove the tibial cutting guide

D. Create cement holes

8. ( ) After completing the posterior condyle resection, the next step usually is:

A. Remove the cutting block

B. Install the tibial trial component

C. Re-check flexion-extension balance

D. Drill cement holes

9. ( ) The first femoral condyle reaming/grinding uses:

A. Size 0 reamer/burr

B. Size 1 reamer/burr

C. Size 2 reamer/burr

D. Trial condyle

10. ( ) Which statement about osteophyte removal is correct?

A. Performed only once, just before the end of the surgery

B. Performed only before measuring the flexion gap

C. Repeated after each reaming/grinding step

D. Performed only after drilling cement holes

11. ( ) After inserting the metal trial component, the first thing to assess is:

A. Intramedullary rod depth

B. Flexion gap

C. Skin incision length

D. Cement hole position

12. ( ) After measuring the flexion gap, the next step is:

A. Re-fix the lower limb

B. Measure the extension gap

C. Install the final prosthesis

D. Remove osteophytes

13. ( ) If secondary reaming/grinding is required, the thickness for further bone removal is calculated based on:

A. Extension gap minus flexion gap

B. Flexion gap minus extension gap

C. Tibial plateau thickness minus femoral trial thickness

D. Cement hole depth minus insert thickness

14. ( ) The instrument used after secondary reaming/grinding is:

A. Curved osteotome

B. Rotary burr

C. T-handle retractor/hook

D. Slap hammer

15. ( ) After installing and securing the tibial trial component, the next critical step is:

A. Use sagittal saw to cut bone and create a keel slot

B. Re-determine the femoral size

C. Immediately install the final prosthesis

D. Perform manual canal opening

16. ( ) The main purpose of the sagittal saw bone cutting stage is:

A. Enlarge the skin incision

B. Create a keel slot for subsequent matching and implantation

C. Drill cement holes

D. Remove the intramedullary rod

17. ( ) After re-checking flexion-extension gap balance, the next step is usually:

A. Prepare/finish the femur

B. Install the final tibial plateau component

C. Re-perform tibial resection

D. End the surgery

18. ( ) After preparing/finishing the femur, the more commonly used instrument for removing residual osteophytes is:

A. Curved osteotome

B. Small oscillating saw

C. Intramedullary rod

D. Metal trial component

19. ( ) After inserting the appropriate size metal trial component, trial condyle, and tibial plateau trial insert, the next step is:

A. Drill cement holes

B. Remove the cutting block

C. Re-hold the ankle/foot

D. Insert the intramedullary rod

20. ( ) Which pairing of accessory instrument and primary use is correct?

A. Z-shaped retractor --- Removing the femoral part

B. T-handle hook --- Exposing the surgical field

C. Slap hammer --- Tool for removing the femoral part

D. Curved osteotome --- Holding the ankle/foot for fixation

**Part II: Fill-in-the-Blank Questions (20 questions, 2 points each, total 40 points)**

1. In the surgical workflow, the step immediately following "Hold the ankle/foot" is _______________.

2. The key operation completed before removing the tibial cutting guide is tibial _______________.

3. After completing the tibial resection, it is necessary to remove the tibial cutting _______________.

4. The step before manual canal opening is femoral _______________.

5. After inserting the intramedullary rod, it is necessary to complete femoral drilling and _______________.

6. To facilitate the removal of the cutting block, it is necessary to first complete posterior condyle _______________.

7. After posterior condyle resection, it is necessary to remove the cutting _______________.

8. After removing the cutting block, the femoral condyle requires initial _______________.

9. After inserting the metal trial component, measure the flexion _______________.

10. After measuring the flexion gap, it is also necessary to measure the _______________ knee gap.

11. Before using the sagittal saw for bone cutting, it is necessary to first install and secure the tibial _______________.

12. During the sagittal saw bone cutting process, besides cutting bone, it is also necessary to create a keel _______________.

13. Before preparing/finishing the femur, it is necessary to re-check _______________-extension gap balance.

14. After re-checking flexion-extension gap balance, the next operation is to prepare/finish the _______________.

15. After preparing/finishing the femur, use a curved osteotome to remove _______________.

16. During the trial phase, insert the appropriate size metal trial component, trial condyle, and tibial plateau trial _______________.

17. Before installing the final prosthesis, it is necessary to first drill cement _______________.

18. After completing cement hole preparation, install the final _______________.

19. The accessory instrument used for exposing the surgical field is the "_______________-shaped retractor".

20. The tool for removing the tibial part is the _______________ hook, and the tool for removing the femoral part is the _______________.
