# Supplementary material for: Serious Game–Assisted Teaching for Junior Operating Room Nurses in Unicompartmental Knee Arthroplasty: Quasi-Historical Controlled Trial
Source: JMIR Serious Games. 2026 Jul 14;14:e93169. doi: 10.2196/93169 (PMC13367756; doi:10.2196/93169)
Supplement: Multimedia Appendix 2 [file games-v14-e93169-s002.docx]

**Multimedia Appendix 1.** Serious Game Design, Workflow, and Representative Screenshots

Overview: The serious game (“Joint Replacement Master”) was developed as a WeChat mini-program to supplement conventional UKA nursing training. The game was designed to support repeated rehearsal of procedural steps in a low-risk environment.

Game structure: The game contains 29 sequential levels corresponding to key stages of the UKA workflow, including instrument recognition, procedural sequencing.

Core interaction: At each level, trainees review a simulated surgical scenario and select the appropriate instrument or action from a virtual tool library. Correct selections allow progression to the next step, whereas incorrect selections trigger immediate feedback and require retry.

Gamification features: The mini-program includes milestone rewards, a ranking mechanism, and audiovisual cues intended to sustain learner interest and encourage repeated use.

Potential analytics: The game can technically record session counts, completion time, and error patterns. These backend usage metrics were not analyzed in the present study.

Figure A1: Level selection interface of the serious game.


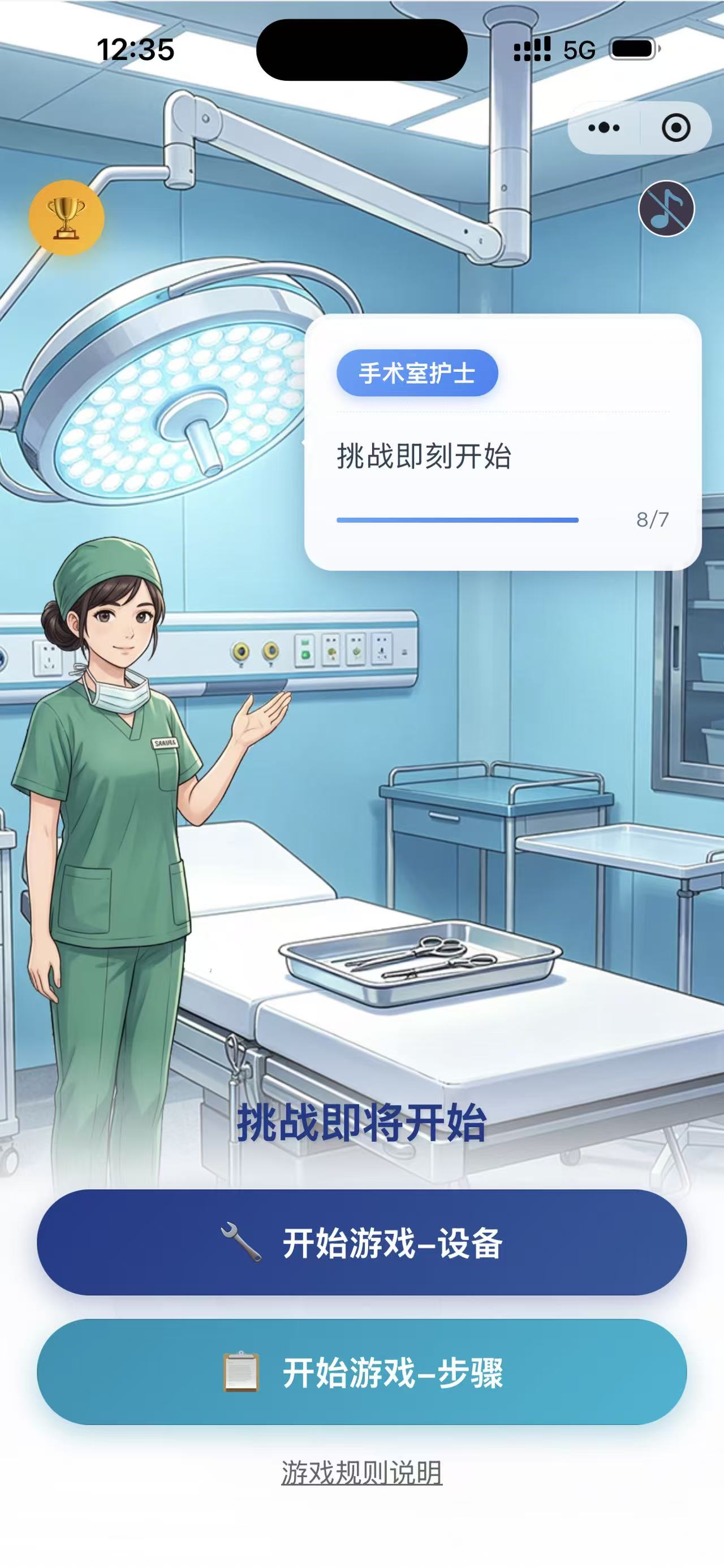


Figure A2: Instrument selection interface during a simulated UKA step.


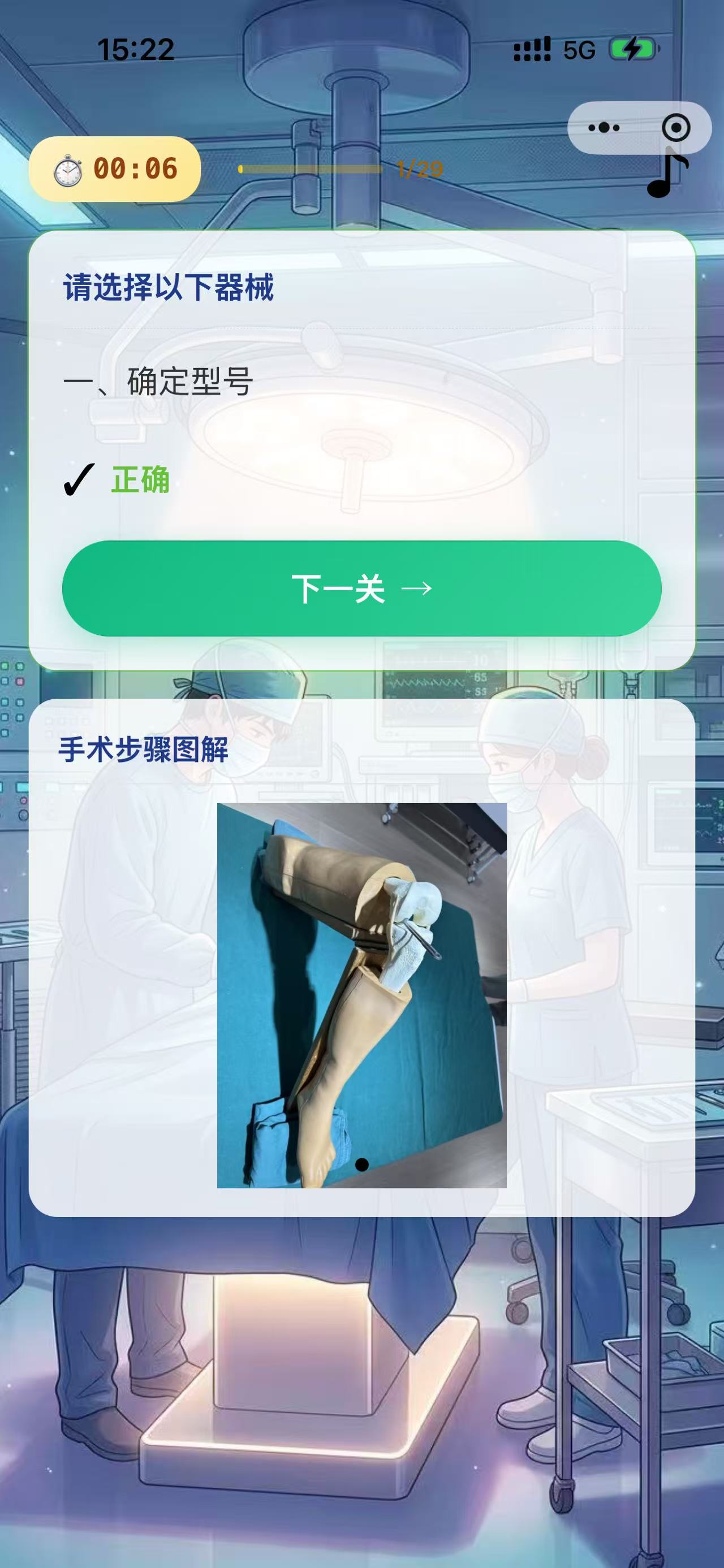


Figure A3: Feedback or reward screen after completion of a game level.


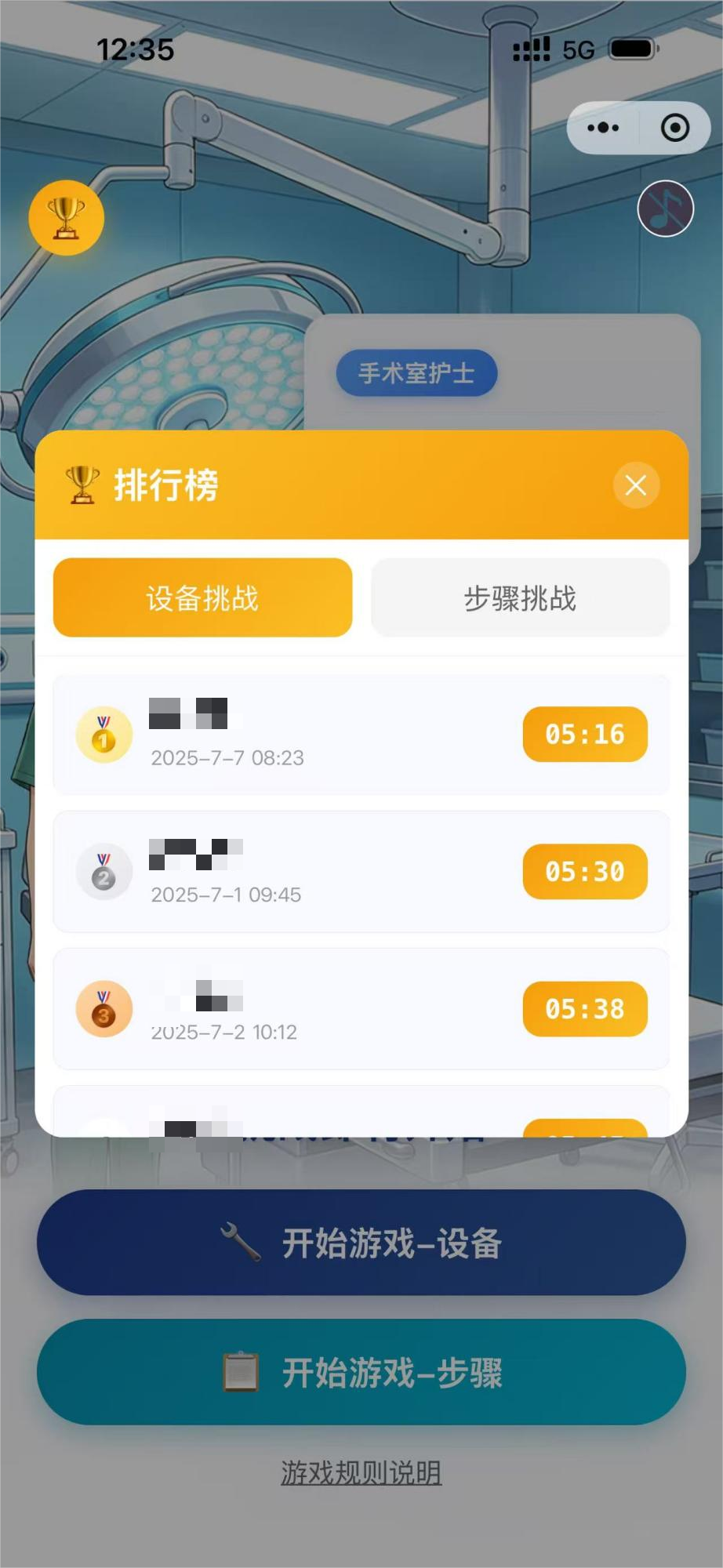


Storyboard of game workflow: Login → level selection → scenario presentation → instrument/action selection → immediate feedback → retry or progression → completion summary.
